# Zooming-in for climate action—hyperlocal greenhouse gas data for mitigation action?

**DOI:** 10.1007/s44168-022-00007-4

**Published:** 2022-04-08

**Authors:** M Jungmann, S N Vardag, F Kutzner, F Keppler, M Schmidt, N Aeschbach, U Gerhard, A Zipf, S Lautenbach, A Siegmund, T Goeschl, A Butz

**Affiliations:** 1 Heidelberg Center for the Environment (HCE), Heidelberg University, Heidelberg, Germany; 2 Institute of Political Science, Heidelberg University, Heidelberg, Germany; 3 Heidelberg, Germany; 4 Private University Seeburg, Seekirchen am Wallersee, Austria; 5 Institute of Earth Sciences, Heidelberg University, Heidelberg, Germany; 6 Institute of Geography, Heidelberg University, Heidelberg, Germany; 7 Research Center for Environmental Economics, Heidelberg University, Heidelberg, Germany

**Keywords:** Climate action, Mitigation, Climate change, Knowledge-action gap, Interdisciplinary, Polycentricity, Climate-change mitigation

## Abstract

While the international community has made progress in adopting goals and agreements in the field of climate change mitigation, efforts to reduce greenhouse gas (GHG) emissions are significantly lacking behind global ambitions for acceptable climate change. In this perspective, we discuss whether a window of opportunity for more effective climate action is emerging due to the convergence of new scientific and technological opportunities to provide high-resolution information on GHG emissions and emerging polycentric governance forms. We hypothesize that scientific and technological developments in the geophysical sciences and geoinformatics could provide the information policy makers need to put in place effective policies on climate change mitigation and to have measures to verify the effectiveness of their mitigation policies. To contribute to a better understanding of these developments and the requirements for effective climate action, new forms of inter- and transdisciplinary research become urgently necessary.

## Introduction

While international climate governance has led to the adoption of pivotal frameworks and global emission reduction targets, such as the Paris Agreement in 2015, and consequently received most media attention, a wide variety of scientific reports, as for instance the Intergovernmental Panel on Climate Change (IPCC)’s sixth assessment report, make clear that humanity is still lagging behind in turning knowledge about climate change into effective mitigation action (Allan et al. 2021; Knutti 2019; Olhoff and Christensen 2020). Partly as a response, polycentric climate governance structures have started to emerge since at least 30 years (Jordan et al. 2015). Polycentricity refers to the emergence of various dynamic and multi-leveled governance forms that include stronger bottom-up elements than traditional international governance forms (Ostrom 2010). Importantly, these new forms of governance do not compete with international governing units, such as the Conference of the Parties (COP), but rather complement each other in a non-hierarchical manner (Tosun and Rinscheid 2021). First sub-national collaboration platforms have started to emerge in the 1990s. City networks and other polycentric forms of governance, such as high-level global forums (Tosun and Rinscheid 2021), were established to enable joint learning experiences and innovation processes. To accelerate the implementation of effective mitigation measures, citizens and decision-makers alike have set high expectations for city-level climate action (Wolfram et al. 2019; Van Der Heijden et al. 2019). According to UN HABITAT, between 71 and 76% of global CO_2_ emissions from global final energy use are produced in urban areas (UN HABITAT 2021). As a consequence, the research community has investigated if and how the high hopes in cities as catalysts for climate action have been met with appropriate actions (Bulkeley and Betsill 2005; 2013). Many scholars came to the conclusion that a diffusion process of learning from other sub-national entities is pivotal for effective climate action and enables sparking innovation and transformation processes among a network (Kern 2019; Jörgensen et al. 2015; Wolfram et al. 2019). Analyzing how local innovation processes for climate mitigation may be triggered and supported is therefore vital to maximize the effect of city-level climate action.

**Fig. 1:**
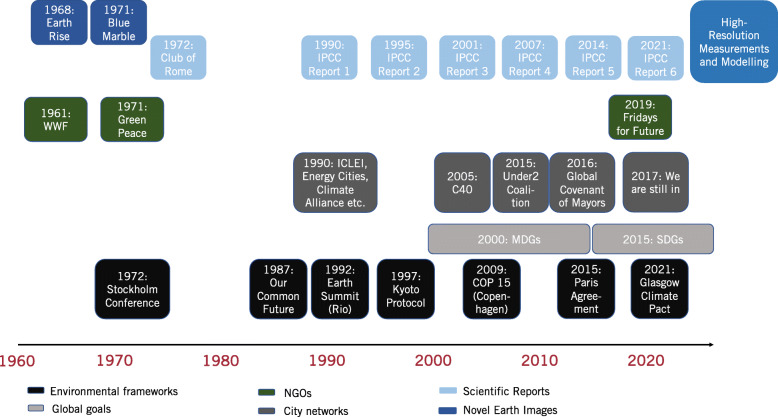
The evolution of international and polycentric climate governance

As Fig. 1 illustrates, after groundbreaking scientific findings on anthropogenic climate change and first calls to action in the 1960s and 1970s, which led to the rise and institutionalization of environmental movements, from 1990 on, major city networks started to evolve. With ICLEI – Local Governments for Sustainability, Energy Cities, the Climate Alliance, and others, the first wave of transnational municipal climate networks started. It was followed by the second wave, which began in the early 2000s and entailed the establishment of C40, the Global Covenant of Mayors, and sub-national initiatives, such as the Under2Coalition, the WeAreStillIn movement, and others. According to Fuhr et al. (2018) the main supporting factors for strengthened local climate action, innovation processes, and policy diffusion through city and sub-national networks, are high problem pressure and capacities to act, democratic structures and processes, enabling policy frameworks, a socio-economic environment that seeks stronger climate action, and leadership through strong individuals, such as dedicated mayors. Despite the fact that many cities across the world have taken a leadership position in climate action, they still receive less public attention than international negotiations and much more decisive action needs to take place in cities to harness their full sustainability potential.

While a variety of factors can be considered as potential reasons for the persisting bias in media attention towards coverage of international forms of climate governance, this media bias may also be caused by how GHG data has been measured and modeled. Following the argumentation of Mol (2006), new information and communication methods have set the stage for a “new informational mode of environmental governance”, in which “environmental information gains transformative powers”. Although more information on national GHG inventories and global stocktaking initiatives exists and thus supports the national and international level decision-making processes, the adequate resolution for local and sub-national decisions on climate action was missing (Hermwille et al. 2019). As a consequence, city networks have not yet unleashed their full potential regarding significant emission reductions on a path towards the achievement of the Paris Agreement. As a form of networked climate governance, they require social innovation and learning to catalyze effective climate action (Tosun and Schoenefeld 2017). To effectively do so, cities require data that matches their relevant scale of action. Policy makers have to find the most effective solutions for the given challenge and although the institutionalization of city networks and other forms of polycentric climate governance may enable cities to learn from each others’ successes and failures, the effectiveness of each governing unit’s climate action as well as the collective innovation power will remain limited, if it is not matched with the respective scale of GHG emission and concentration data.

In this paper, we argue that the asymmetry that has existed in terms of the scales of GHG emissions data may be on the brink of disappearing and the required high-resolution data to facilitate effective climate action will be provided within the next years. Scientific and technological developments in the geophysical sciences and geoinformatics can provide the information policy makers need to put in place effective policies on climate change mitigation and to have measures to verify the effectiveness of their mitigation policies. High-resolution greenhouse gas information, visualization, and models hold the potential to enable opportunity structures, learning experiences, and innovation processes that cities can use to tailor their policies to most effectively mitigate emission sources (Van Der Heijden et al. 2019). We hypothesize that this rise of high-resolution data could decisively contribute to a narrowing of the “knowledge-action gap” for policymakers in climate change because of an unprecedented level of nuance in GHG emissions data that meets the demands of societal and political actors and enables them to boost effective climate action. Whether this hypothesis holds true, remains to be tested, but numerous developments indicate that a significant change in perception and action is feasible. In recent years, the measurement and modeling community has embarked on building multi-faceted GHG observing systems with the goal of providing policy-relevant emissions information (Ciais et al. 2014; Mueller et al. 2021). Against the backdrop of these developments, we explore how to optimize information provision for effective climate action.

Similar to the vast new opportunities high-resolution GHG emission measurements and modeling hold for effective political climate action, changes in individual perceptions and behaviors may become possible. Evidence on whether more information can impact on individual behavior (Bolderdijk et al. 2013) is mixed. Yet, we argue, the nature of the information is key. Action is least likely for problems described as distal, affecting the actor with low likelihood and high uncertainty and that are not brought about by a clear cause. We propose that “zooming-in” will improve on these typical aspects of climate communications. It will foster effective climate action by strengthening climate protection norms via creating an awareness of consequences of, responsibility for and control over GHG emissions that global-scale measurements have not been able to establish (Stern 2000). Awareness of consequences and a sense of responsibility, on the one hand, capture the fact that specific changes in the environment become attributable to identifiable human activities on the political, administrative, and political level. A sense of control, on the other hand, can be fostered through hyperlocal GHG emissions data, as improvements in environmental outcomes become attributable to identifiable mitigation actions in a direct, relatable, and intuitive way (Klöckner 2013). Furthermore, high-resolution information will necessarily improve prioritization of the most effective measures (Marghetis et al. 2019). Beyond the individual actor, providing highly resolved pollution information has been shown to contribute to emissions reductions through market interactions (Barwick et al. 2019), political processes (Wang et al. 2021), and the legal system (Gray and Shimshack 2011). Evidence from studies on toxic release inventories and other environmental issues has shown that the disclosure of environmental information can impact individual (Loewenstein et al. 2014), community level (Fung and O’rourke 2000), market level (Mastromonaco 2015) and regional level (Overdevest 2005) decisions on environmental challenges. It does not follow that similar effects will be accomplished through high-resolution GHG data. It makes the conjecture sufficiently plausible, however, to merit further investigation.

## The rise of high-resolution greenhouse gas data

The Keeling curve recording atmospheric CO_2_ concentrations since the 1950s at Mauna Loa, Hawaii, has become the icon for a range of networks that track global anthropogenic GHG emissions as well as the continental-scale processes of the constituent cycles. Coarse resolution satellite observations of CO_2_ and CH_4_ have been available for about 20 years (Butz et al. 2011). Now, after a phase of technological consolidation, they are about to transition into operational missions that can contribute to the regular GHG stocktakes foreseen by the Paris agreement. An example is the Copernicus CO_2_ Monitoring (CO2M) constellation to be launched into orbit by the mid-2020s. Together, the networks and satellites form the backbone of a GHG observing system (Ciais et al. 2014) that prioritizes global, continental, and national scales. These developments are paralleled at the neighborhood- and facility-scale at which there is rapid technological progress towards assessing GHG emissions (Christen 2014). For example, building on a dense network of tens of low-cost CO_2_ sensors in the California Bay Area, the BEACO_2_N (BErkeley Atmospheric CO_2_ Observation Network) was able to quantify and map, with neighborhood resolution, the CO_2_ emissions reductions related to the mobility restrictions during the COVID-19 lockdown in spring 2020 (Turner et al. 2020). Urban flux measurements allow for mapping sector specific CO_2_ emissions (Stagakis et al. 2019). New satellite and airborne sensors are able to image the instantaneous CO_2_ and CH_4_ plumes emanating from coal-fired power plants and oil and gas production sites with resolutions of meters to a few tens of meters (Duren et al. 2019).

**Fig. 2:**
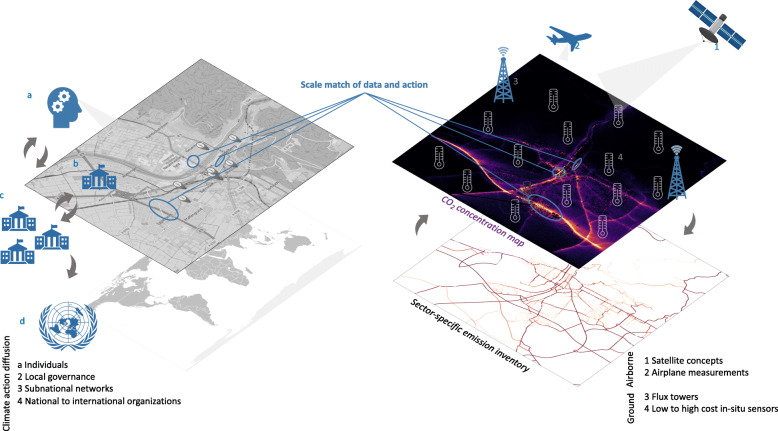
The rise of a suite of observations such as satellite concepts (1), airplane measurements (2), flux towers (3) and low-cost as well as high-cost sensors (4) enables a scale match of data and action. This can lead to a new awareness of hyperlocal emission mitigation potential as well as emission validation possibilities and thus, can trigger climate action by **a** citizens and **b** local governance, which can further diffuse within **c** sub-national networks and **d** national to international organizations

Modeling studies are following the focus on local scales as the respective measurements become available for comparison (Lauvaux et al. 2020; Turner et al. 2020) and as computational power now enables high-resolution simulations for longer time periods. The model developments are mirrored by progress in inventory making, which traditionally collects emissions on the national scale, but new techniques use downscaling methods to smaller areas of interest. While downscaling introduces larger uncertainties (Mueller et al. 2021), its implementation is becoming more sophisticated and pilot emission inventories such as HESTIA are able to provide high-resolution estimates of greenhouse gases for pilot cities (Gurney et al. 2019). With these new advances in quality, coverage and diversity of measurements, models and inventories (see Fig. 2), foundations are now in place to locate verified CO_2_ and CH_4_ emissions on an unprecedented “hyperlocal” resolution promising source attribution on facility and neighborhood scale.

Despite these new technological opportunities, some key challenges on the path towards a large-scale practical application of high-resolution GHG information remain in terms of sensor-handling, data fusion and interpretation. On the one hand, the calibration of measurements remains challenging and requires a thorough handling and filtering of the raw data, comparison to other measurements and careful assessment of uncertainties. On the other hand, the interpretation of the measured signals is especially challenging in areas in which spatial and temporal variability occurs due to specific local contexts. Sensor network design as well as model approaches need to take such heterogeneity into account. Finally, more practical challenges, such as finding social and political support to finance and install appropriate scientific sensor networks currently hamper replicating these new technological advances at a large scale. To harness the full potential of new technological opportunities regarding emission measurements and modeling, a user-centric approach needs to be adopted and tailoring data to the actual needs of different stakeholders will be essential. How such data then impacts perception patterns, behaviors, and decision-making opens up various new questions for social science research within the field of climate mitigation.

## A new era for the social science of climate action?

Despite the large amount of research on the knowledge-action gap, the effects of high-resolution GHG emissions data on climate action have only recently started to garner attention in the social sciences, with a focus on citizen science, household studies, real-world labs, and other transdisciplinary research (Diederich and Goeschl 2018; Knutti 2019). We present three new perspectives for social science research on climate change mitigation, which have evolved and will likely continue to receive significant attention by the research community in years to come.

First, the developments regarding the technical opportunities to measure, model, and visualize GHG information on a hyperlocal scale directly raise the question of which data to measure and how to aggregate needs steering to prevent investments that do not instigate change in relevant stakeholders such as cities. To understand which kind of data is actually needed and has significant effects on mitigation actions is of both societal and academic relevance. Such research would directly connect with the large amount of publications on urban climate governance, especially in terms of how effective local climate policies can be designed and how transformation processes can be enhanced (Kern 2019; Van Der Heijden et al. 2019; Bulkeley 2021; Jörgensen et al. 2015).

Second, this provides new opportunities for social science research to investigate the potential effects of such data on individual, societal, and political climate action and interdependence between the respective units. Hyperlocal GHG data provides potential for individuals and civil society to attribute responsibility to the lowest levels of the politico-administrative system and to mobilize for climate action privately or through political processes, legal institutions, or market processes. The locus of attribution suggests several dimensions for transdisciplinary research, especially since this institutional setting allows for better observing the actions and reactions of policymakers (Lahsen et al. 2020). Sociologists are well versed to investigate which civil society groups make use of hyperlocal GHG data and for what purpose, and how they present this kind of data to reach the widest possible audiences. This highlights the fundamental role of mutual communication for triggering action. Civil society tends to combine the strategy of approaching policymakers with reaching out to the media and attempting to create public attention (Dür and Mateo 2013). Psychologists, behavioral economists and experts in political participation can explain under what conditions such mobilization attempts succeed and how individuals react to hyperlocal GHG data. Do they demand action from policymakers? Do they change their own behavior? Turning to the perspective of local policymakers and administrations, GHG data that allows for identifying variation at the district- or street-level can provide a strong incentive to strengthen climate action. Political scientists can illuminate whether GHG emissions become more politicized and entail the adoption of more effective policy instruments. The use of measurement devices and data by citizens then can produce a situation in which the monitoring of such measures is carried out by volunteers, which can result in various types of responses by these and other individuals that can be best understood by applying theories and concepts from (behavioral) economics, (political) psychology, and (political) sociology. The potential feedback effects of new policies and procedures adopted by policymakers, NGOs and civil society is another line of research that will benefit from the involvement of various disciplines but led by sociologists.

Third, we need rigorous impact assessment for what is changed with the provision of high-resolution GHG information. Recent changes regarding climate governance, in particular the move towards a polycentric system and strengthened national and international climate pledges signal increased ambition to mitigate GHG emissions (Jordan et al. 2015; Bulkeley 2021; Bulkeley and Betsill 2013). Thus, it remains to be investigated whether and how high-resolution emissions information can catalyze the efforts of new forms of collaboration and innovation on path towards the achievement of the 1.5 ^∘^C goal. Data-based solutions for effective local climate action have the potential to spark innovation in other cities through city networks (Kern 2019; Jörgensen et al. 2015; Wolfram et al. 2019). At the same time solutions may diffuse to higher and lower level administration such as the state or individual level (Fuhr et al. 2018). How such diffusion processes work and how local and sub-national collaboration enables innovation processes requires further research. At the same time, it is clear that they have the potential to exponentiate the impact of local climate action and may scale such mitigation effects. Therefore, informed and effective decisions on strengthened climate action, based on high-resolution GHG emissions information holds the potential to allow the scaling of local solutions through polycentric systems to global advancements regarding emission reductions (Fuhr et al. 2018; Kern 2019). To effectively scale up best practices from the local level, however, a matching of scales on the data level is needed to bridge the currently prevailing knowledge-action gap in climate change mitigation.

## Zooming-in for climate action

At this time, we seek to establish the perspective that high-resolution GHG emissions and concentration information, if utilized and communicated effectively, could provide the link between public awareness and local climate action that was previously missing. We believe that the idea of “zooming-in for climate action” merits investigation. One reason is evidence that awareness of climate change has led to some, albeit insufficient, global-scale action. Some have credited the publication of the “Blue Marble” pictures with founding the environmental movement and facilitating the institutionalization of international organizations, such as the UNFCCC, the Kyoto Protocol, the 2030 Agenda for Sustainable Development, and ultimately the Paris Agreement. This makes it plausible, at least, that—at the other end of scales—local awareness can lead to local action. A second reason is that climate change governance is increasingly moving from an international to a polycentric system. At the same time, societal pressure, through organizations such as Fridays for Future, has drastically increased and already influenced political agendas, for instance in the context of green recovery measures after the economic downturn due to the COVID-19 pandemic. More granular data provides societal groups ammunition to demonstrate that district-level climate action can be improved. A missing component for polycentric governance is access to high-resolution GHG information that meets the local and sub-national scales of political and individual action with its implicit notions of source attribution, responsibility, and actionability.

A rigorous test of whether high-resolution GHG data can set into motion climate action, will require a new form of inter- and transdisciplinary research. Many factors influence how decision-makers, whether individual or societal, seek and process information and how this translates into motivation for climate action. Quantity and quality of available data, trust in science, and communication types and channels are only the most obvious. Transdisciplinary research helps to better understand societal perceptions and demands and establish a trustful relationship between academia and society. As a consequence, with regards to the highly complex and interconnected challenges posed by climate change and the increasingly short amount of time for humanity to effectively mitigate GHG emissions to meet climate goals, a new form of research needs to evolve that investigates the demand of citizens and politics for science-based, high-resolution GHG data (in terms of both, the time and geographical dimension), contributes to better information for individuals and decision-makers to base their decisions on and analyze how such information affects their perceptions and actions.

For different disciplines and sectors to effectively collaborate, the proper working environment needs to be established. Past experiences with interdisciplinary projects have shown that the success of true interdisciplinary cooperation, which goes beyond individual researchers continuing to work within their silos and occasionally merging research results in joint publications or proposals, requires well established networks and regular forms of exchange where researchers trust each other, develop a common language, and have communication channels that allow them to quickly and effectively develop and test new hypotheses (Brewer 1999; Lélé and Norgaard 2005). To facilitate the development of such inter- and transdisciplinary networks, which is urgently needed considering the time left for effective climate action, the research landscape needs to change. We argue that incentive and reward systems for inter- and transdisciplinary research need to evolve to enable and promote efforts to go beyond disciplinary boundaries (Bromham et al. 2016). Such initiatives are already challenging in themselves since it requires researchers to leave their disciplinary comfort zones. The present research landscape, however, further discourages researchers from taking this step since career options and reputation are still largely based on disciplinary publications and awards (Szell et al. 2018), and research funding opportunities are mostly structured along disciplinary boundaries (Bromham et al. 2016). To change this, more inter- and transdisciplinary grants need to be established and scholars, researchers, reviewers etc. need to be trained early on to develop a true interdisciplinary mindset that starts with the development of joint research questions and designs, while taking into account societal and environmental needs and changes. Moreover, programs for early-career interdisciplinary researchers need to enable them to develop their own scientific identity while receiving excellent training in disciplinary and interdisciplinary approaches and methods (Haider et al. 2018).

At the same time, hyperlocal data may foster a cooperative scientific culture in the field of climate action science, since the outlined potential of hyperlocal data requires effective inter- and transdisciplinary research to unfold. Such research could transform the existing understanding of informational environmental governance and enable individuals and decision-makers alike to take more effective climate action.

## Data Availability

All data and material relevant for this paper has been included in the manuscript.
